# Supplementary material for: Effects of Variation in Urine Sample Storage Conditions on 16S Urogenital Microbiome Analyses
Source: mSystems. 2022 Dec 8;8(1):e01029-22. doi: 10.1128/msystems.01029-22 (PMC9948722; doi:10.1128/msystems.01029-22)
Supplement: TEXT S1 [file msystems.01029-22-s0001.docx]

**Text S1 – Supplemental Materials and Methods**

1. Volunteer Recruitment and Sample Collection

Volunteers were recruited via word of mouth at the UC San Diego Health Women's Pelvic Medicine Center, focusing on a healthy adult female population. Our inclusion criteria for “healthy” was defined by the participant being free of active symptoms of disease such as urinary tract infection, fever or chills, cough, shortness of breath, headache, loss of taste or smell, sore throat, congestion, nausea or vomiting, and diarrhea. After being provided with and reading through a written document, volunteers gave verbal consent under the IRB-approved study (UCSD protocol #801735) before sample donation. A waiver of written consent was given to this study, as the only risk to study participation was loss of confidentiality. Verbal consent only allowed for further preservation of confidentiality and improved risk/benefit ratio.

Ten healthy adult females donated a single 10mL urine sample using the Peezy Midstream (Forte Medical) collection device. After sample donation, urine aliquots of 500uL were immediately transferred at room temperature from the Peezy collection tube into 15mL screw cap tubes (Sarstedt, Inc.) that contained a 1:10 volume of Genelock (Sierra Molecular) to urine or no Genelock. Immediately after aliquoting the samples, the samples were stored in their respective temperature condition outlined in **Figure 1A,** and transported to the lab. When transporting the samples to the lab, the following protocols were followed: samples needing to be initially stored at 4°C (4°C and heat cycle samples) were transported on ice in styrofoam coolers; samples needing to be initially stored at -20°C (-20°C and freeze-thaw samples) were transported on dry ice in styrofoam containers; finally, samples that needed to be kept at ambient temperature were transported in a container with ambient temperature being controlled at ~21-23°C. Urine samples included 3 technical replicates per temperature condition. Genelock-only (containing no urine sample) samples were used as negative controls and included 2 technical replicates per temperature condition.

Once transported to the lab, the samples were stored in the following fashion: samples initially stored at 4°C were stored in the fridge, samples initially stored at -20°C were stored in the -20°C freezer, and samples initially stored at ambient temperature were stored in a cabinet in the lab at ~23°C. Samples that underwent the 4°C condition, -20°C condition, and ambient condition were kept in these respective conditions for 48 hours. Samples that underwent the freeze-thaw condition underwent two rounds of freeze-thaw in the following fashion: the samples were initially stored at -20°C, moved to ambient temperature at 12 hours post-collection, moved back into -20°C at 24 hours post-collection, and moved back to ambient temperature at 36 hours post-collection. Samples that underwent the heat cycle condition underwent two rounds of heat cycle in the following fashion: the samples were initially stored at 4°C, moved to 40°C at 12 hours post-collection, moved back into 4°C at 24 hours post-collection, and moved back to 40°C at 36 hours post-collection. At the end of 48 hours, all samples were stored in a -80°C freezer until sample processing. Pictographic detailing can be found in **Figure 1A**.

1. KatharoSeq isolate growing

The KatharoSeq positive controls used to establish the limit of detection were *Bacillus subtilis* and *Paracoccus denitrificans*. *B. subtilis*, a Gram positive bacterium, and *P. dentrificans*, a Gram negative bacterium, are commonly found in the soil and not typically in human microbiome samples, making them suitable controls. The two strains were grown independently at 37°C, and harvested at the log stage. Serial dilutions of the media were made while in the log stage, and 100uL of each dilution was plated onto an LB agar plate and incubated overnight at 37°C. The number of cells on each plate were counted using standard microbiology methods, and plates that contained between 10-100 cells were further extrapolated. The two strains were mixed together to act as one control at a cell count of 1.91 x 10^8 cells per mL. Further information regarding KatharoSeq protocol can be found in Minich, et al. (1).

1. Plating and Extraction

Urine samples and Genelock-only negative controls were taken out of -80°C storage and thawed on ice before centrifuging for 1 min at 10,000 RCF. 200uL of the sample pellet was aliquoted into a 96-well MagMAX Microbiome Bead Plate (ThermoFisher Scientific) via pipetting. Eight positive controls containing a 10-fold serially diluted mock community (*Paracoccus denitrificans* and *Bacillus subtilis*) were plated with cell known cell ranges from 248,300 to 0 cells, and eight extraction blanks (no sample) were included as negative controls. Once samples and controls were plated, the samples were extracted using the MagMAX Microbiome Ultra NA Extraction Kit (ThermoFisher Scientific). All samples were plated and extracted using Earth Microbiome Project standard protocols ([https://earthmicrobiome.org/protocols-and-standards/)](https://earthmicrobiome.org/protocols-and-standards/), as updated in Shaffer, et al. [(2).](https://www.zotero.org/google-docs/?sYI6I3)

1. 16S rRNA Amplification and Sequencing

After extraction, the 16S rRNA V4 region was amplified using unique 515f-806r Golay barcodes

[(https://earthmicrobiome.org/protocols-and-standards/16s/)](https://earthmicrobiome.org/protocols-and-standards/16s/) to allow post-sequencing demultiplexing. Miniaturized amplicon PCR reactions were used, as outlined in Minich, et al.

[(3),](https://www.zotero.org/google-docs/?8n7sLy) to lower overall reagent cost and save gDNA and primer. Post-PCR 16S rRNA libraries were equal volume pooled and PCR cleaned using the QIAquick PCR Purification Kit (QIAGEN). Cleaned library pools were then sequenced on an Illumina MiSeq using a MiSeq 300 cycle (2x151 paired end reads) reagent kit (v2). All 16S rRNA amplification and sequencing protocols adhered to Earth Microbiome Project standard protocol ([https://earthmicrobiome.org/protocolsand-standards/)](https://earthmicrobiome.org/protocols-and-standards/), as updated in Minich, et al (3).

1. Post-Sequencing Processing

After sequencing, forward read sequences generated from the MiSeq were trimmed to 150 nucleotides, quality filtered, and demultiplexed using Qiita (Qiita study 14383, EBI accession ERP138439) [(4).](https://www.zotero.org/google-docs/?26w6w8) We utilized the 50% KatharoSeq threshold to exclude and rarefy samples to 986 reads, resulting in a final analysis pool of 9 participants for a total of 161 samples. One participant had a read count below the 50% KatharoSeq threshold, prompting us to exclude their data.

**References:**

1. Minich, J. J., Zhu, Q., Janssen, S., Hendrickson, R., Amir, A., Vetter, R., Hyde, J., Doty, M. M., Stillwell, K., Benardini, J., Kim, J. H., Allen, E. E., Venkateswaran, K., & Knight, R. (2018). Katharoseq enables high-throughput microbiome analysis from low-biomass samples. *mSystems*, *3*(3), e00218-17. https://doi.org/10.1128/mSystems.00218-17
2. Shaffer, J. P., Marotz, C., Belda-Ferre, P., Martino, C., Wandro, S., Estaki, M., Salido, R.

A., Carpenter, C. S., Zaramela, L. S., Minich, J. J., Bryant, M., Sanders, K., Fraraccio, S., Ackermann, G., Humphrey, G., Swafford, A. D., Miller-Montgomery, S., & Knight, R. (2021). A comparison of DNA/RNA extraction protocols for high-throughput sequencing of microbial communities. *BioTechniques*, *70*(3), 149–159. https://doi.org/10.2144/btn2020-0153

1. Minich, J. J., Humphrey, G., Benitez, R. A. S., Sanders, J., Swafford, A., Allen, E. E., & Knight, R. (2018). High-throughput miniaturized 16s rrna amplicon library preparation reduces costs while preserving microbiome integrity. *mSystems*, *3*(6), e00166-18. https://doi.org/10.1128/mSystems.00166-18
2. Gonzalez, A., Navas-Molina, J. A., Kosciolek, T., McDonald, D., Vázquez-Baeza, Y.,

Ackermann, G., DeReus, J., Janssen, S., Swafford, A. D., Orchanian, S. B., Sanders, J.

G., Shorenstein, J., Holste, H., Petrus, S., Robbins-Pianka, A., Brislawn, C. J., Wang, M.,

Rideout, J. R., Bolyen, E., … Knight, R. (2018). Qiita: Rapid, web-enabled microbiome meta-analysis. *Nature Methods*, *15*(10), 796–798. https://doi.org/10.1038/s41592-018-

0141-9
